# Post-RT Head and Neck DCE-MRI: Association Between Mandibular Dose and v_e_

**DOI:** 10.3390/cancers17193224

**Published:** 2025-10-03

**Authors:** Brandon Reber, Renjie He, Moamen R. Abdelaal, Abdallah S. R. Mohamed, Samuel L. Mulder, Laia Humbert Vidan, Clifton D. Fuller, Stephen Y. Lai, Kristy K. Brock

**Affiliations:** 1 Department of Imaging Physics, The University of Texas MD Anderson Cancer Center, Houston, TX 77030, USA; kkbrock@mdanderson.org; 2 Department of Radiation Oncology, Mayo Clinic, Rochester, MN 55905, USA; smulder@mdanderson.org (S.L.M.); lhumbert@mdanderson.org (L.H.V.); 3 Department of Radiation Oncology, The University of Texas MD Anderson Cancer Center, Houston, TX 77030, USA; rhe1@mdanderson.org (R.H.); asmohamed@mdanderson.org (A.S.R.M.); cdfuller@mdanderson.org (C.D.F.); sylai@mdanderson.org (S.Y.L.); 4 Department of Radiation Oncology, Emory University, Atlanta, GA 30322, USA; 5 Department of Head and Neck Surgery, The University of Texas MD Anderson Cancer Center, Houston, TX 77030, USA; 6 Department of Radiation Physics, The University of Texas MD Anderson Cancer Center, Houston, TX 77030, USA

**Keywords:** head and neck cancer, dynamic contrast-enhanced MRI, osteoradionecrosis of the jaw

## Abstract

Background: Dynamic contrast-enhanced magnetic resonance imaging (DCE-MRI) is a functional imaging modality that can quantify tissue permeability and blood flow. Due to vasculature changes resulting from radiation therapy (RT), DCE-MRI quantitative parameters should be significantly different in regions receiving a high radiation dose compared to regions receiving a low radiation dose. This study sought to determine whether a significant difference exists in post-head-and-neck-cancer (HNC)-RT DCE-MRI quantitative parameters (K^trans^ and v_e_) between regions of the mandible receiving a high radiation dose and regions of the mandible receiving a low radiation dose. Methods: DCE-MRI was acquired from HNC subjects post-RT. The DCE-MRI quantitative parameters K^trans^ and v_e_ were obtained through Tofts model fitting. Four mandible sections (left ramus, left body, right ramus, and right body) were delineated on subject mandible contours. Two Friedman tests comparing the mean K^trans^ and v_e_ in low-dose (≤60 Gy) areas of the four mandible regions were computed. If the Friedman test determined that a significant difference for a parameter between mandible regions exists, post hoc Wilcoxon signed-rank tests were completed comparing the four mandible regions. If the Friedman test determined that there was no significant difference between mandible regions, a Wilcoxon signed-rank test was used to determine whether a significant difference exists in the parameter between high-dose (>60 Gy) and low-dose (≤60 Gy) mandible regions. Results: 48 HNC subjects were included in the analysis. The Friedman tests showed no significant difference in v_e_ means between mandible regions ($\chi_{\left(3\right)}^{2}$ = 1.63, *p* = 0.44) and a significant difference in K^trans^ means between mandible regions ($\chi_{\left(3\right)}^{2}$ = 10.29, *p* = 0.005). Post hoc testing between K^trans^ mandible regions found that the left body and right body differed significantly from the left ramus and right ramus. The Wilcoxon signed-rank test comparing the mean v_e_ between high- and low-dose mandible regions found a significant difference (*W* = 214, *p* = 0.00013). Conclusions: no inherent difference in the DCE-MRI quantitative parameter v_e_ was observed within subject mandibles, but a significant difference was observed between v_e_ means in high- and low-radiation-dose mandible regions. These results provide evidence of the utility of DCE-MRI to monitor mandible vasculature changes resulting from head and neck cancer radiation therapy. Monitoring post-HNC-RT mandible vasculature changes is important to initiate earlier toxicity management and ultimately improve HNC survivors’ quality of life.

## Simple Summary

Dynamic contrast-enhanced magnetic resonance imaging (DCE-MRI) can detect relative differences in anatomical blood perfusion and vessel permeability. Differences in vascularity should occur between mandible regions receiving a large radiation dose and regions receiving a low radiation dose during head and neck cancer radiation therapy. In this study, we determined whether DCE-MRI can be used to detect vasculature differences between mandible regions irradiated with high and low amounts of radiation. The results indicate that one of the DCE-MRI parameters, v_e_, was significantly different between irradiated mandible regions receiving high and low radiation dose. This parameter may be used as an early marker for mandible radiation damage from head and neck radiation therapy.

## 1. Introduction

In 2020, over 800,000 patients were diagnosed with head and neck cancer (HNC), and over 400,000 HNC-related deaths occurred worldwide [1]. HNCs form in the mucosal surfaces of the pharynx, larynx, oral cavity, paranasal sinuses, and salivary glands [2]. Risk factors for HNC vary depending on the disease subsite, but they generally include tobacco use, alcohol consumption, human papillomavirus status, and overall oral health [3]. Owing to an increase in HNC associated with human papillomavirus, incidence rates for HNC are expected to increase by 30% from 2018 to 2030 [4].

The most common treatment method for head and neck squamous cell carcinoma is a combination of surgery, chemotherapy, and radiation therapy (RT) [5,6]. The delivered radiation dose depends on several factors, but it typically ranges from 56 Gy to 70 Gy [6]. Several toxic effects, such as xerostomia and dysphagia, can occur during or after RT [7]. Osteoradionecrosis (ORN) is a toxic effect that can result from radiation exposure across the volume of the mandible, especially in the treatment of oral cavity and oropharyngeal cancers [8,9]. Given the cumulative lifetime risk for ORN and the resulting impact on patient function and quality of life, identifying patients at risk of ORN or already suffering from the early stages of ORN is critical [10,11].

Dynamic contrast-enhanced (DCE)-MRI is a functional imaging modality that can measure blood perfusion, vascularity, and permeability in regions of interest [12]. DCE-MRI involves the injection of a contrast agent that alters the measured MRI signal in regions adjacent to the contrast agent [13]. These changes in signal intensity are ultimately related to differing blood perfusion and tissue permeability within the imaged regions [13]. Several pharmacokinetic models are available to determine the physiological relationship to measured signal intensities. One of the most commonly used pharmacokinetic models is the Tofts model [14]. This model defines several quantitative parameters, including K^trans^, the transfer constant from plasma to the extravascular extracellular space (EES), and v_e_, the fractional volume of the EES [14]. According to the Tofts model, differences in these parameters between regions indicate relative differences in tissue permeability and blood perfusion. Because RT-damaged tissues can show changes in blood perfusion and tissue permeability [15,16], DCE-MRI quantitative parameters related to perfusion and tissue permeability should differ among radiation-damaged and non-radiation-damaged tissues.

Previously, researchers have used DCE-MRI for HNC imaging, such as for locoregional recurrence monitoring [13], HNC tumor staging and grading [17], histopathology correlation [18], and treatment response monitoring [19]. Some previous studies have looked at using DCE-MRI to characterize the vasculature and perfusion changes within the mandible resulting from HNC-RT [20,21,22,23]. In one study, investigators examined the changes in DCE-MRI parameters before and after treatment in the mandibles of rabbits and found that DCE-MRI may be able to model maxillofacial wound healing [23]. In another study, researchers compared K^trans^ and v_e_ in regions of the same mandible that did and did not have osteoradionecrosis and found that the K^trans^ and v_e_ were significantly different between ORN-affected and ORN-free regions [22]. Another study demonstrated significant voxel-wise differences in K^trans^ and v_e_ using DCE-MRI before and after RT [20]. However, to date, no study has looked at differences in DCE-MRI parameters in different mandibular regions irradiated with high and low radiation doses not necessarily related to observable ORN.

In this work, we sought to determine whether DCE-MRI can detect changes in the permeability and blood perfusion in the mandible as a result of HNC-RT. To this end, we analyzed the post-RT means of K^trans^ and v_e_ in high-dose mandible regions (>60 Gy) and those in low-dose mandible regions (≤60 Gy). Owing to tissue damage resulting from RT, K^trans^ and v_e_ should differ significantly between the regions because of differences in blood perfusion and tissue permeability.

## 2. Methods

### 2.1. Overview

**Figure 1:**
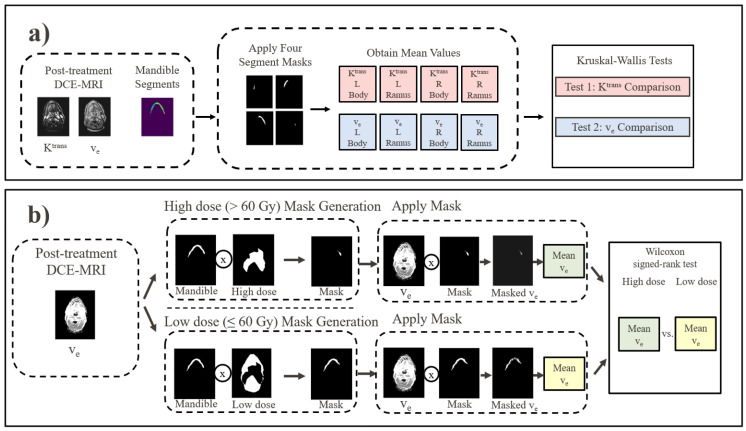
Overview of the research methodology. (**a**) Comparison process between the DCE-MRI of the four mandible regions. This was completed to determine whether an inherent difference in DCE-MRI exists between the four mandible regions unrelated to changes associated with tissue damage from radiation therapy. (**b**) DCE-MRI comparison between high- and low-dose regions of the mandible. This process determines whether the DCE-MRI can capture changes in mandibular K^trans^ and v_e_ associated with radiation therapy. Since the Friedman tests in (**a**) showed that only v_e_ was independent of mandible location, it was the only parameter to be tested in (**b**).

The overall research methodology was split into three parts. First, DCE-MRI quantitative parameters K^trans^ and v_e_ were collected, curated, and registered. Next, the parameters for the low-dose mandible regions were analyzed for inherent significant differences in K^trans^ and v_e_ within the mandible. Finally, the means of K^trans^ and v_e_ in the high-dose mandible regions were compared to parameter means in low-dose mandible regions. The second and third methodology components are summarized in Figure 1.

### 2.2. Patient Cohort

Patients were included from an ongoing clinical trial at the University of Texas MD Anderson Cancer (clinicaltrials.gov ID: NCT03145077). The trial enrollment eligibility criteria were patients older than 18 years, curative RT for HNC, ability to undergo MRI, and an Eastern Cooperative Oncology Group (ECOG) performance status score of 0–2. Patients received standard-of-care prescription doses ranging from 60 Gy to 70 Gy delivered in 30 to 35 fractions. All patients underwent follow-up DCE-MRI at least 1 month after RT completion. Patients were excluded if they received treatment with multiple modalities, such as a combination of intensity-modulated RT and intensity-modulated proton therapy.

### 2.3. Data

Patient RT dose maps, gross tumor volume (GTV) contours, and treatment planning CT scans were acquired from a clinical database in RayStation 11B (RaySearch Laboratories, Stockholm, Sweden). T2-weighted images were acquired using a Siemens Aera 1.5T MRI scanner (Siemens Healthineers, Erlangen, Germany; TE/TR = 80/4800 ms, matrix size = 512 × 512, slice thickness = 2 mm, voxel spacing = 0.5 mm × 0.5 mm, and 1 average) during the post-treatment DCE-MRI (TE/TR = 1.07/5 ms, matrix size = 256 × 208, slice thickness = 4 mm, voxel spacing = 1 mm × 1 mm, 1 average). The contrast agent gadobutrol (Gadovist; Bayer Healthcare, Leverkusen, Germany) was injected with a power injector (Spectris MR Injector; MedRad, Pittsburgh, PA, USA) at a dose of 0.1 mmol/kg body weight at 3 mL/s. In combination with the contrast agent, saline was administered at 3 mL/s at the same quantity as that of the contrast agent. The pharmacokinetic modeling procedure used to fit the DCE-MRI was described previously [24]. Briefly, the Tofts model was used, which can be defined as follows [14]:

$$\frac{dC_{TOI}(t)}{dt}\;=\;K^{trans}\;\times {\;C}_{p}\left(t\right)\;-\;k_{ep}\;\times \;C_{TOI}(t),$$

where $C_{TOI}(t)$ is the concentration of the contrast agent in the tissue of interest in units of mmol/L, $K^{trans}$ is the volume transfer constant of the contrast agent from the plasma into the EES in units of min^−1^, $C_{p}(t)$ is the contrast agent in the plasma in units of mmol/L, and $k_{ep}$ is the rate constant of contrast agent from the EES back to the plasma in units of min^−1^. $k_{ep}$ is related to the volume fraction of the EES through the following formula:

$$k_{ep}\;=\;\frac{K^{trans}}{v_{e}}$$

DCE-MRI relies on tracking the concentration of the contrast agent over time. However, the MRI scanner measures signal intensity (SI), not concentration. Converting SI to concentration requires knowledge of the baseline tissue state, specifically, the longitudinal relaxation time (*T*_10_) before the contrast agent arrives. We converted SI (S(t)/S_0_, S_0_ is baseline) to concentration based on the spoiled gradient echo sequence (SPGR) signal equation (assuming a steady state and ignoring *T*_2_* effects):

$$C\left(t\right)=\frac{1}{TR\cdot r_{1}}\ln\left(\frac{1\;-\;\frac{S\left(t\right)}{S_{0}}\cos\theta \left(\frac{1\;-\;e^{-\frac{TR}{T_{10}}}}{1\;-\;e^{-\frac{TR}{T_{10}}}\cos\theta }\right)}{1\;-\;\frac{S\left(t\right)}{S_{0}}\left(\frac{1\;-\;e^{-\frac{TR}{T_{10}}}}{1\;-\;e^{-\frac{TR}{T_{10}}}\cos\theta }\right)}\right)\;-\;\frac{1}{T_{10}\cdot r_{1}}$$

Here, θ is the flipping angle, TR is the repetition time, and r_1_ is the relaxivity of the contrast agent. We measured T_10_ for each voxel immediately before the DCE acquisition sequence. This was achieved using a separate, fast T_1_-mapping sequence such as inversion recovery, Look–Locker, and variable flip angle (VFA). Each technique has its own advantages and disadvantages, and the choice of technique depends on the specific application and the available imaging hardware. We used VFA with SPGR for T_10_ map acquisition.

B_1_ correction should be employed for a dedicated T_1_ biomarker to avoid B_1_ variance across different setups and measurements. Because the T_1_ maps were acquired as part of the DCE protocol, B_1_ variation has less impact than for a single DCE acquisition. Therefore, B_1_ correction was not necessary on the 1.5T systems.

The arterial input function (AIF) was obtained on a per-patient basis. For each subject, a region of interest (ROI) was manually placed in the external carotid artery, identified on the pre-contrast T1-weighted images. AIF curves were then fitted with a validated 7-parameter biexponential, bilinear model as described by Parker et al. [25] to mitigate the effects of noise and partial volume averaging.

Motion and noise are two primary challenges in obtaining reliable, quantitative DCE-MRIs. Our previous research study [24] describes the management of these artifacts as employed here. Our method is a principled, optimization-based reconstruction framework that uses a rigorous mathematical framework to find the most physiologically plausible, clean image series that could have generated the noisy, motion-corrupted data that were acquired. This results in more reliable and robust extraction of pharmacokinetic parameters such as $K^{trans}$ and $v_{e}$ compared to the use of simple filters alone.

### 2.4. Registration

Rigid registration was performed to align each patient’s DCE-MRI and treatment dose maps. The DCE-MRI map was in the same frame of reference as that of the T2-weighted image, and the treatment dose map was in the same frame of reference of the treatment planning CT. The rigid registration was completed between the patient’s T2-weighted and treatment planning CT images. The T2-weighted image was used as the fixed image, and the CT image was used as the moving image. The intensity-based rigid registration was completed using RayStation 11B. A mandible contour of the head and neck was generated on each patient’s treatment planning CT image using an atlas-based segmentation in RayStation 11B. SimpleITK (version 2.3.0) was then used to resample the CT image, dose map, GTV contour, and mandible contour to the spacing of the DCE-MRI parameters. For the CT image and dose map, linear resampling was completed. For the binary masks, nearest-neighbor resampling was completed. The resulting outputs were the treatment planning CT, mandible contour, GTV contour, and dose images that were registered and resampled to the K^trans^ and v_e_ images.

### 2.5. Comparison of DCE-MRI in Different Mandible Regions

Regions of interest in the mandible corresponding to the left ramus, left body, right body, and right ramus were delineated by a graduate student on each patient’s treatment planning CT map. Next, voxels were removed from the K^trans^ and v_e_ regions of the mandible that corresponded to either high-dose (>60 Gy) or GTV regions. These voxels were removed to test inherent DCE-MRI differences between mandible regions while limiting the potential effect of high dose or the GTV on DCE-MRI parameters. The mean values of voxels within each of the four regions of interest for each patient were then collected for the K^trans^ and v_e_ parameters separately. In all, eight mean values were collected for each patient: K^trans^ left ramus, K^trans^ left body, K^trans^ right body, K^trans^ right ramus, v_e_ left ramus, v_e_ left body, v_e_ right body, and v_e_ right ramus (Figure 1a).

### 2.6. High- and Low-Dose Volume Selection

After DCE-MRI comparisons between mandible regions, separate additional comparisons were completed between DCE-MRI high-dose (>60 Gy) and low-dose (≤60 Gy) mandible regions. The dose threshold of 60 Gy was chosen due to prior evidence of mandibular doses > 60 Gy being associated with an increased ORN risk [26]. Two binary dose masks were created using patient dose masks to select the high-dose and low-dose regions of the mandible. The GTV portions of the mandible were removed from the masks. The GTV was removed from DCE-MRI masks to limit the effects of DCE-MRI changes resulting from the GTV. The means of the high- and low-dose volumes were then computed for both the K^trans^ and v_e_ parameters separately. In all, four mean values were generated for each patient: high-dose K^trans^, low-dose K^trans^, high-dose v_e_, and low-dose v_e_ (Figure 1b).

### 2.7. Statistical Analysis

Three sets of statistical tests were completed. The first set of tests compared the low-dose areas of the four mandible regions to determine whether an inherent significant difference in DCE-MRI exists within the mandible. This set of tests comprised two Friedman tests. One test compared the K^trans^ of the four mandible groups and the other test compared the v_e_ of the four mandible groups. If a Friedman test for a parameter showed a significant difference in the DCE-MRI parameter between anatomical regions, a set of post hoc Wilcoxon signed-rank tests comparing the four anatomical regions was completed for that parameter. Post hoc Wilcoxon signed-rank tests were completed for K^trans^ due to the Friedman test showing a significant difference in the parameter between anatomical regions. Next, a third set of tests was completed to determine if a significant difference exists in DCE-MRI parameters between the high- and low-dose DCE-MRI regions. This set of tests comprised Wilcoxon signed-rank tests, which compare per patient DCE-MRI differences. This test was only completed if the Friedman test for the DCE-MRI parameter showed no significant difference between anatomical regions. Due to the K^trans^ parameter showing a significant difference in the parameter between anatomical regions, only the v_e_ parameter was tested. This Wilcoxon signed-rank test compared the high- and low-dose v_e_ groups. A Bonferroni correction was applied to address multiple comparisons for all tests. The first family of tests, the Friedman tests, had an adjusted significance level of $\alpha =\frac{0.05}{2}=0.025$. The second family of tests, the post hoc Wilcoxon signed-rank tests for K^trans^, had an adjusted significance level of $\alpha =\frac{0.05}{6}=0.008$. Finally, the third family of tests, the Wilcoxon signed-rank test comparing high- and low-dose v_e_, had an adjusted significance level of $\alpha =\frac{0.05}{1}=0.05$.

## 3. Results

In total, 48 subjects were included in the study. A summary of the 48 patients’ demographics is presented in Table 1.

**Figure 2:**
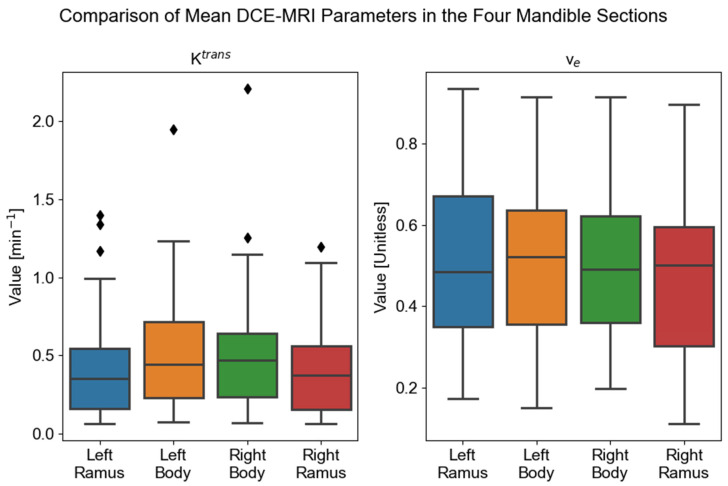
Comparison of DCE-MRI between mandible regions. The Friedman test comparing K^trans^ means between mandible regions found a significant difference between regions (*p* < 0.025). The Friedman test comparing v_e_ means between mandible regions found no significant difference between regions (*p* > 0.025). Diamonds in the K^trans^ plot correspond to data points that have values larger than Q_3_+1.5*IQR.

Boxplots of the K^trans^ and v_e_ values for the left ramus, left body, right body, and right ramus are shown in Figure 2. We found a significant difference between mandible regions for the K^trans^ parameter ($\chi_{\left(3\right)}^{2}$ = 10.29, *p* = 0.005). We did not find a significant difference between mandible regions for the v_e_ parameter ($\chi_{\left(3\right)}^{2}$ = 1.63, *p* = 0.44).

We completed post hoc testing for the K^trans^ parameter due to the Friedman test results. Six Wilcoxon signed-rank tests were completed. The left ramus showed significant differences with the left body (*W* = 229, *Z =* −3.54, *p* = 0.0004) and right body (*W* = 227, *Z =* −3.57, *p* = 0.0004), but not with the right ramus (*W* = 548, *Z =* −0.17, *p* = 0.87). The left body showed a significant difference with the right ramus (*W* = 346, *Z =* −2.48, *p* = 0.013), but no significant difference with the right body (*W* = 535, *Z =* −0.54, *p* = 0.59). Finally, the right body showed a significant difference with the right ramus (*W* = 328, *Z =* −2.67, *p* = 0.0077).

**Figure 3:**
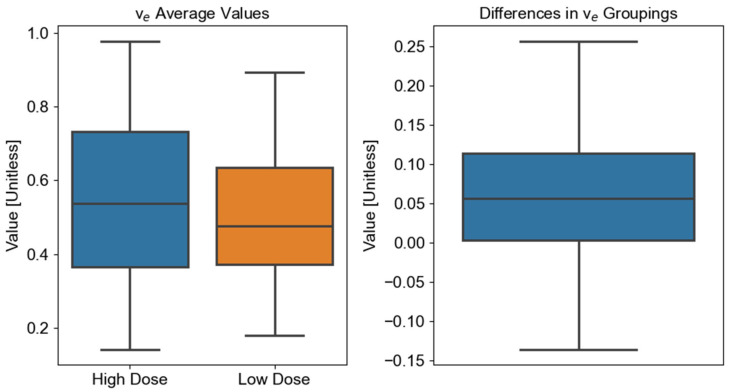
Summary of the v_e_ comparison between the high- and low-dose mandible regions. The left boxplot shows the distribution of the calculated mean v_e_ values in the images. The high-dose box corresponds to the mean of v_e_ in mandible regions that received >60 Gy. The low-dose box corresponds to the mean of v_e_ values in mandible regions that received ≤60 Gy. The boxplot on the right shows the v_e_ differences between the high- and low-dose regions in the images. The v_e_ differences are computed between the high- and low-dose regions for the same patient. There was a significant difference in v_e_ between high- and low-dose regions (*p* < 0.05).

Finally, the Wilcoxon signed-rank test comparing the differences in v_e_ means between the high-dose and low-dose regions determined that the two regions were significantly different (*W* = 214, *Z =* 3.85, *p* = 0.00013) (Figure 3).

## 4. Discussion

The goal of this study was to determine whether DCE-MRI can be used as an imaging biomarker for detecting mandibular physiological changes associated with high radiation doses from head and neck cancer radiation therapy. The results show that there was an inherent significant difference in K^trans^ between mandible regions not attributed to a high amount of delivered radiation, but there was no significant difference for v_e_. Next, the high- and low-dose regions of the mandible were compared to determine whether there were significant differences between the DCE-MRI of the two regions. Due to the results of the Friedman test, only v_e_ was tested. A statistically significant difference between the high- and low-dose regions of the mandible were identified for the v_e_ parameter. This parameter could be potentially used to identify radiation damage or osteoradionecrosis development within the mandible earlier, compared to when symptoms present clinically, allowing for earlier management of treatment-related symptoms.

The results of the Friedman test were different between the two DCE-MRI variables studied: the K^trans^ parameter was significantly different between mandible regions unrelated to a large delivered radiation dose whereas the v_e_ parameter was not significantly different between mandible regions. If the measured K^trans^ and v_e_ correspond to the physiological parameters specified by the Tofts model, the results indicate that different mandible regions have differing permeability but similar EES.

The post hoc Wilcoxon signed-rank tests comparing K^trans^ means between mandible regions provide evidence that the left and right mandible bodies have different K^trans^ values compared to the left and right ramus. This suggests that the mandible anatomical site is more important for K^trans^ values compared to laterality. The results suggest that mandible bodies are more vascularized compared to the rami, leading to larger K^trans^ values in mandible bodies.

The significant difference in v_e_ between high- and low-dose regions provides evidence of the capability of v_e_ as a radiation biomarker to detect mandibular anatomical and physiological changes related to radiation therapy. Radiation therapy can cause a variety of changes in mandibles post-irradiation, such as inflammation and altered permeability [27,28,29,30]. The results suggest that changes to the EES in tissue caused by radiation, such as fibrosis and cell density, can be detected by measurable changes in v_e_ post radiation therapy.

Several choices were made regarding the registration and resampling of the images. In this study, a rigid registration was used to register the T2w image and the CT instead of a deformable image registration. The overall purpose of the image registration was to align the DCE-MRI images to the treatment dose, specifically in the mandibular area. This approach was deemed appropriate due to minimal deformation in bone structures such as the mandible. Next, the treatment dose spacing was resampled to the spacing of the DCE-MRI instead of resampling the DCE-MRI spacing to the treatment dose spacing. This was completed because the dose was used only as a binary mask whereas the DCE-MRI values were used in the analysis.

This study has several limitations. First, there might be some uncertainty between the DCE-MRI and treatment plan registration. If there is a slight misregistration, the continuous nature of the dose maps should limit the impact on the results. Next, a single delineation of the mandible regions was completed for the mandible DCE-MRI comparisons. For this comparison, the consistency of the delineation of mandible regions is most important. Although the mandible region delineations were completed as accurately as possible, the results showing no inherent differences between mandible regions should remain if consistent slight delineation deviations from actual mandible structures exist. Dental artifacts present an additional challenge to DCE-MRI studies. Metal can cause local susceptibility artifacts that can potentially lead to signal distortion. Although we avoided including cases with extensive distortion, dental artifacts have the potential to impact pharmacokinetic parameters. Finally, a single dose (60 Gy) was used to distinguish between high- and low-dose regions. This dose was chosen due to it being at the lower end of the common HNC-RT prescription range of around 60–70 Gy in addition to 60 Gy being a potential threshold for ORN development [26,31].

The work presented here complements other studies investigating the relationship between radiation dose and DCE-MRI in the mandible [20,21,22,23]. In a study conducted with a rabbit cohort, the change in K^trans^ and v_e_ of the mandible pre-radiation compared to post-radiation had no significant difference in the parameters between a control group that was not irradiated versus an experimental group that was irradiated [23]. However, there was a significant difference in DCE-MRI parameter changes between rabbits that received a mandible surgical procedure post-radiation compared to rabbits that received a mandible surgical procedure without prior radiation [23]. In comparison to this study, our work examined human post-treatment DCE-MRI rather than rabbit changes in DCE-MRI pre- and post-RT. Another study completed an analysis looking at the voxel-wise changes in DCE-MRI pre-RT and post-RT and found a significant difference in both the K^trans^ and v_e_ parameters [20]. Our study examined post-RT images rather than the change between pre-RT and post-RT and looked at differences between mandible regions rather than voxel-wise changes [20]. Finally, one study compared K^trans^ and v_e_ parameters of radiation therapy associated with osteoradionecrosis (ORN)-affected regions of the mandible to control regions on the opposite side of the mandible [22]. It was found that there was a significant difference in K^trans^ and v_e_ in ORN-affected volumes compared to the contralateral control volumes [22]. In addition, no correlation was found between the mean dose, min dose, max dose, and dose delivered to 95% of the ORN-ROIs between different subjects [21]. In comparison to that study, this study examined DCE-MRI differences within each subject’s mandible between high- and low-dose regions and not DCE-MRI differences between ORN+ and ORN- affected regions [22]. Although not all regions that receive a large radiation dose will develop ORN, several studies have shown that the delivery of a high dose to mandibular regions is a risk factor for ORN development [32,33,34].

This research provides evidence that changes in DCE parameters post-therapy may serve as a response biomarker [35]. Future efforts are underway to formalize biomarker assessment as a monitoring biomarker of mandibular radiation injury, leading to consequential ORN in observational cohorts [36]. By establishing a dichotomized (high/low) dose-response in a pilot cohort, this study provides justification for further efforts focused on the construction of imaging biomarker-informed normal tissue complication probability models that incorporate DCE MRI metrics.

## 5. Conclusions

This study investigated whether a significant difference in post-RT DCE-MRI quantitative parameters exists between regions of the mandible receiving high and low radiation doses. After determining that the DCE-MRI quantitative parameter v_e_ did not differ based on anatomical location, a significant difference in v_e_ means was observed between regions of the mandible that received a high and low radiation dose. Determining whether a significant difference in these parameters exists for regions at risk for developing ORN due to radiation damage may motivate the development and validation of clinically relevant imaging-based biomarkers. Evaluating alterations in DCE-MRI parameters as a surrogate for radiation damage could identify patients at risk for ORN, allowing for earlier treatment interventions for ORN and related toxicities associated with head and neck cancer radiation therapy.

## Figures and Tables

**Table 1:** Patient demographic characteristics (n = 48).

Characteristic	n (%)
Median age, years	64 (IQR: 13)
Male sex	43 (90%)
Current smoker	1 (2%)
Former smoker	28 (58%)
Median packs per year, n	
Current smoker	18 (IQR: 0)
Current and former smokers	15 (IQR: 12.5)
Tumor site	
Oral cavity	6 (13%)
Oropharynx	40 (83%)
Other *	2 (4%)
HPV associated	35 (73%)
Cancer Staging	
Stage I	19 (40%)
Stage II	10 (21%)
Stage III	4 (8%)
Stage IV	7 (15%)
Unknown/unspecified	8 (17%)

* Other tumor sites were the hypopharynx, larynx, nasopharynx, and unknown.

## Data Availability

In accordance with NOT-OD-21-013, Final NIH Policy for Data Management and Sharing, anonymized/de-identified data that support the findings of this study are openly available in an NIH-supported generalist scientific data repository (figshare) at https://doi.org/10.6084/m9.figshare.29627075.v1, https://doi.org/10.6084/m9.figshare.29646260.v1.
